# Comparative study of transcatheter aortic valve implantation versus conventional surgical aortic valve replacement in the treatment of severe aortic stenosis with reverse ventricular remodeling

**DOI:** 10.3389/fcvm.2025.1712400

**Published:** 2026-01-06

**Authors:** Haoyan Li, Sumin Yang, Wenlong Yan, Xiaodong Chen, Xun Chi

**Affiliations:** Cardiovascular Surgery, The Affiliated Hospital of Qingdao University, Qingdao, China

**Keywords:** SAVR - surgical aortic valve replacement, TAVR - transcatheter aortic valve replacement, aortic stenosis (AS), reverse ventricular remodeling, echocardiography

## Abstract

**Introduction:**

This study aimed to compare echocardiographic outcomes and analyze the changes in ventricular remodeling at different time points after surgery in patients with severe aortic stenosis (AS) undergoing either surgical aortic valve replacement (SAVR) or transcatheter aortic valve replacement (TAVR).

**Methods:**

This retrospective study consecutively enrolled 175 patients with severe AS who underwent either SAVR or TAVR. Transthoracic echocardiograms obtained at baseline, 30 days, and 1 year after the procedure were analyzed by multiple echocardiographers at our institution.

**Results:**

At baseline, the TAVR group was significantly older (74 ± 7 years vs. 62 ± 9 years, *p* < 0.001) and had a higher prevalence of hypertension (53.5% vs. 31.0%, *p* = 0.003), coronary artery disease (38.4% vs. 23.0%, *p* = 0.028), and atrial fibrillation (16.3% vs. 2.3%, *p* = 0.002). Additionally, the TAVR cohort demonstrated significantly worse cardiac functional status (*p* < 0.001). Compared to TAVR patients, SAVR patients (*N* = 87) exhibited a more pronounced reduction in left ventricular end-systolic dimension (−0.5 ± 0.65 cm vs. −0.2 ± 0.47 cm, *p* < 0.001) and left ventricular end-diastolic dimension (−0.6 ± 0.64 cm vs. −0.3 ± 0.55 cm, *p* < 0.001) at the 1-month follow-up. A decrease in left ventricular mass was observed in both groups from baseline to 1 month postoperatively, with the SAVR group showing a significantly greater reduction (LV mass: −67.3 ± 59.31 g vs. −38.2 ± 46.49 g, *p* = 0.003; LVMI: −39.1 ± 33.93 g/m^2^ vs. −22.5 ± 27.08 g/m^2^, *p* = 0.005). However, these differences were not sustained at the 1-year follow-up. SAVR patients experienced a transient decline in right ventricular function at 1 month, which recovered by 1 year postoperatively. At the 1-year follow-up, the TAVR group experienced a higher incidence of Major Adverse Cardiac Events (MACE) (*p* = 0.01), despite showing significant improvement in the severity of both mitral and tricuspid regurgitation compared to baseline (*p* < 0.001). Although pulmonary artery pressure improved in both groups after AVR, the SAVR group demonstrated significantly lower pressure at 1 year (*p* < 0.001).

**Conclusion:**

In patients with severe aortic stenosis, SAVR was associated with more significant regression of left ventricular dimensions and mass at 1 month compared to TAVR, alongside a transient impairment of right ventricular function. By 1 year postoperatively, however, no significant differences in ventricular remodeling were observed between the two groups.

## 1 Introduction

Aortic stenosis (AS) is a common primary valvular disease worldwide, particularly in developed countries. With the aging of the population, its prevalence continues to rise (1). Without intervention, symptomatic aortic stenosis significantly increases the risk of death due to heart failure or angina (2). The only effective treatment for severe AS is aortic valve replacement (AVR). Previous randomized trials have demonstrated that transcatheter aortic valve replacement (TAVR) is superior or non-inferior to surgical aortic valve replacement (SAVR) in patients with severe AS across various surgical risk profiles, establishing it as a valuable alternative (3–6).

Post-AVR left ventricular reverse remodeling is a major determinant of clinical symptoms and outcomes (7). Consequently, comparing ventricular reverse remodeling after TAVR vs. SAVR has become a focus of recent research (8). Prior randomized controlled trials have preliminarily identified differences between TAVR and SAVR in terms of aortic valve hemodynamics, left ventricular remodeling, right heart functional changes, and postoperative valvular regurgitation (9, 10). However, findings regarding certain ventricular parameters have been inconsistent.

Moreover, reverse ventricular remodeling is a relatively prolonged process. Notably, a meta-analysis by F. Sousa Nunes et al. first reported significant LV reverse remodeling as early as 1 month after AVR (8). Nevertheless, whether the most pronounced remodeling occurs within this early postoperative period remains uncertain. Echocardiography plays a key role in evaluating the evolution of cardiac geometry and function after AVR, as well as assessing the hemodynamic performance and stability of prosthetic valves.

This study aims to compare echocardiographic outcomes in patients with severe AS following SAVR or TAVR, analyze differences in aortic valve hemodynamics, left ventricular remodeling, and right heart functional changes, and determine whether there are statistically significant differences in ventricular remodeling at various postoperative time points.

## 2 Methods

### 2.1 Study design and population

**Figure 1:**
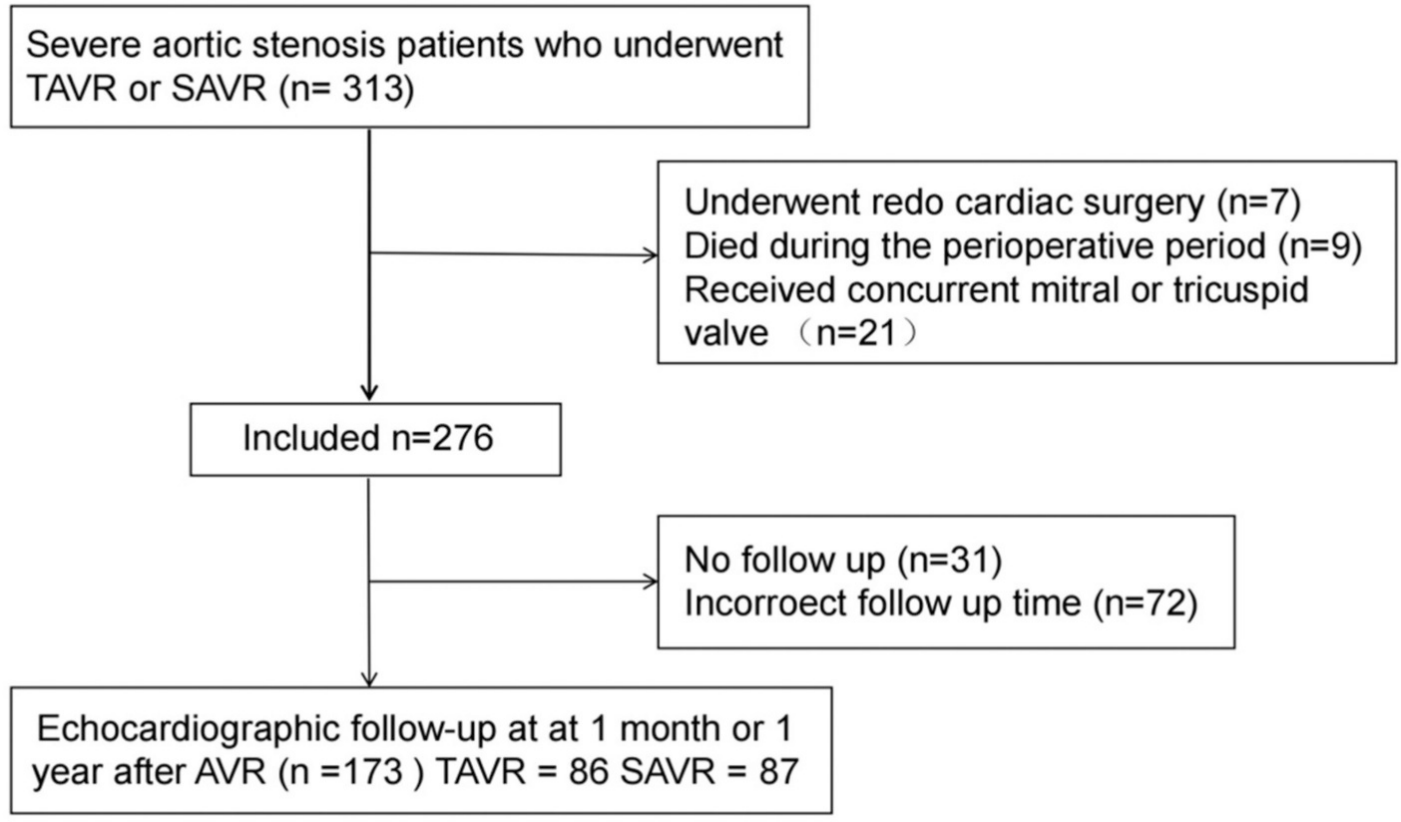
Patient sample. TAVR, transcatheter aortic valve replacement; SAVR, surgical aortic valve replacement; AVR, aortic valve replacement.

This retrospective study consecutively enrolled 313 adult patients with severe aortic stenosis who underwent either transcatheter or surgical aortic valve replacement (TAVR or SAVR) at the Department of Cardiovascular Surgery, The Affiliated Hospital of Qingdao University, between January 2019 and February 2024. Patients who underwent redo cardiac surgery, died during the perioperative period, or received concurrent mitral or tricuspid valve replacement/repair due to severe valvular disease were excluded (*n* = 37, see Figure 1). Additionally, we excluded patients lost to follow-up (*n* = 31) and those who did not undergo echocardiographic examinations at the recommended postoperative time points (*n* = 72). A total of 173 patients were included in the final analysis: 86 in the TAVR group and 87 in the SAVR group. Among them, 5 TAVR patients underwent concurrent percutaneous coronary intervention (balloon angioplasty and stenting), while 19 SAVR patients received coronary artery bypass grafting, and 4 SAVR patients underwent concomitant aortic root surgery. Baseline assessments included demographic characteristics, comorbidities, medication regimens, clinical symptoms, laboratory results, and transthoracic echocardiographic data. The primary clinical endpoint was the incidence of major adverse cardiac events (MACE) at 1 month and 1 year postoperatively, which was a composite of cardiovascular mortality, non-fatal myocardial infarction, non-fatal stroke, and rehospitalization for heart failure.

Secondary outcomes included the degree of aortic paravalvular regurgitation, changes in pulmonary artery pressure, and improvements in regurgitation of other valves (assessed by transthoracic echocardiography).

The study protocol was approved by the Ethics Committee of The Affiliated Hospital of Qingdao University. All data were anonymized, and involved no commercial interests or additional interventions. Given the retrospective nature of the study and use of de-identified data, informed consent was waived.

### 2.2 Procedure

In the TAVR group, the procedure was performed via the femoral artery approach in 84 cases and via the transapical approach in 3 cases. Balloon-expandable valves were implanted in 85 patients, while self-expanding valves were used in 2 patients. Pre-dilation (balloon aortic valvuloplasty before TAVR) and post-dilation (after TAVR) were performed at the operator's discretion. The heart team routinely performed coronary CT angiography for all patients scheduled to undergo TAVR. If severe coronary artery disease was confirmed, percutaneous coronary intervention (PCI) was performed prior to aortic valve replacement.

76 patients in the SAVR group underwent biological valve replacement, while other 11 patients received mechanical valve replacement. Similarly, patients with significant coronary artery disease underwent concomitant coronary artery bypass grafting (CABG), with the specific sequence of procedures determined at the operator's discretion.

### 2.3 Echocardiography analyses

Transthoracic echocardiographic data were collected at baseline, at 1 month, and at 1 year postoperatively. Main parameters measured included: peak aortic gradient, mean aortic gradient, effective orifice area (EOA), left ventricular end-diastolic dimension (LVEDD), left ventricular end-systolic dimension (LVESD), interventricular septal thickness (IVS), left ventricular posterior wall thickness (LVPW), and tricuspid annular plane systolic excursion (TAPSE). The effective orifice area was calculated using the continuity equation (11, 12). Left ventricular mass and mass index (LVMI) were determined in accordance with the American Society of Echocardiography M-mode criteria (13).We defined more pronounced left ventricular reverse remodeling as the maintenance of a normal left ventricular mass index at follow-up (≤95 g/m^2^ for women and ≤115 g/m^2^ for men). Left ventricular ejection fraction was assessed using the Simpson's biplane method.

### 2.4 Statistical analyses

Statistical analyses were performed using SPSS (version 27.0.1) and R (version 4.2.2). Continuous variables were expressed as mean (standard deviation) for normally distributed data and median (interquartile range) for non-normally distributed data, while categorical variables were summarized as frequencies and percentages. Normality was assessed using normality tests and Q-Q plots, with appropriate descriptive statistics applied based on the distribution. Group comparisons for normally distributed continuous variables were conducted using Welch's *t*-test or ANOVA, whereas the Wilcoxon rank-sum test or Kruskal–Wallis test was used for non-normally distributed variables. Categorical variables between the treatment groups were compared using Fisher's exact test or the chi-square test, as appropriate. Changes in echocardiographic parameters following transcatheter or surgical aortic valve replacement (TAVR/SAVR) were analyzed using multivariable linear regression models adjusted for potential covariates.

## 3 Results

### 3.1 Baseline findings

Table 1 presents the baseline characteristics stratified by procedure group (TAVR, *n* = 86; SAVR, *n* = 87). Compared with the SAVR group, the TAVR group was significantly older (74 ± 7 years vs. 62 ± 9 years, *p* < 0.001) and had a higher prevalence of hypertension (53.5% vs. 31.0%, *p* = 0.003), coronary artery disease (38.4% vs. 23.0%, *p* = 0.028), and atrial fibrillation (16.3% vs. 2.3%, *p* = 0.002). Additionally, the TAVR cohort exhibited significantly worse cardiac functional status (*p* < 0.001), with a higher proportion of patients in NYHA class III (62.8% vs. 55.2%) and class IV (12.8% vs. 2.3%). No significant differences were observed between the groups in terms of sex distribution, body mass index (BMI), pacemaker implantation status, aortic valve morphology, severity of valvular regurgitation, preoperative NT-proBNP levels, or other underlying comorbidities.

**Table 1:** Patient demographics and baseline characteristics.

Characteristic	Surgery		*p*-value
	TAVR *N* = 86	SAVR *N* = 87	
Sex, *n* (%)			
Male	53 (61.6%)	52 (59.8%)	0.802
Female	33 (38.4%)	35 (40.2%)	
Age, Mean ± SD	74 ± 7	62 ± 9	<0.001
BMI, Mean ± SD	24.5 ± 3.7	25.4 ± 3.9	0.135
Pacemaker carrier, *n* (%)			
Yes	6 (7.0%)	2 (2.3%)	0.168
No	80 (93.0%)	85 (97.7%)	
Main symptom, *n* (%)			
Asymptomatic	7 (8.1%)	2 (2.3%)	0.120
Dyspnea	64 (74.4%)	59 (67.8%)	
Angina	10 (11.6%)	18 (20.7%)	
Syncope	5 (5.8%)	8 (9.2%)	
NYHA, *n* (%)			
Ⅰ	7 (8.1%)	2 (2.3%)	<0.001
Ⅱ	14 (16.3%)	35 (40.2%)	
Ⅲ	54 (62.8%)	48 (55.2%)	
Ⅳ	11 (12.8%)	2 (2.3%)	
AV type, *n* (%)			
Tricuspid	72 (83.7%)	74 (85.1%)	0.809
Bicuspid	14 (16.3%)	13 (14.9%)	
AV regurgitation, *n* (%)			
Mild	52 (60.5%)	53 (60.9%)	0.951
Moderate	34 (39.5%)	34 (39.1%)	
MV regurgitation, *n* (%)			
Mild	71 (82.6%)	78 (89.7%)	0.177
Moderate	15 (17.4%)	9 (10.3%)	
TV regurgitation, *n* (%)			
Mild	75 (87.2%)	83 (95.4%)	0.056
Moderate	11 (12.8%)	4 (4.6%)	
NT-proBNP, Median (Q1, Q3)	1,284 (402, 2,839)	946 (596, 2,338)	0.482
Hypertension, *n* (%)			
Yes	46 (53.5%)	27 (31.0%)	0.003
No	40 (46.5%)	60 (69.0%)	
Diabetes, *n* (%)			
Yes	28 (32.6%)	17 (19.5%)	0.051
No	58 (67.4%)	70 (80.5%)	
CAD, *n* (%)			
Yes	33 (38.4%)	20 (23.0%)	0.028
No	53 (61.6%)	67 (77.0%)	
AF, *n* (%)			
Yes	14 (16.3%)	2 (2.3%)	0.002
No	72 (83.7%)	85 (97.7%)	
Hyperlipidemia, *n* (%)			
Yes	33 (38.4%)	34 (39.1%)	0.924
No	53 (61.6%)	53 (60.9%)	
COPD, *n* (%)			
Yes	2 (2.3%)	1 (1.1%)	0.621
No	84 (97.7%)	86 (98.9%)	

BMI, body mass index; NYHA, New York Heart Association functional class; AV, aortic valve; MV, mitral valve; TV, tricuspid valve; CAD, coronary artery disease; COPD, chronic obstructive pulmonary disease; AF, atrial fibrillation.

As shown in Table 2, significant differences were also noted in baseline hemodynamic and echocardiographic parameters, including peak aortic jet velocity (4.75 ± 0.57 m/s vs. 5.02 ± 0.68 m/s, *p* = 0.005), peak transaortic pressure gradient (91.77 ± 23.01 mmHg vs. 102.79 ± 28.77 mmHg, *p* = 0.006), and mean pressure gradient (55.70 ± 16.32 mmHg vs. 61.43 ± 18.10 mmHg, *p* = 0.030). In contrast, baseline left ventricular dimensions, left ventricular mass, ejection fraction, and right ventricular function were comparable between the two groups.

**Table 2:** Baseline echocardiographic results.

Variable	TAVR (*n* = 86)	SAVR (*n* = 87)	*P*-value
Peak velocity, m/s	4.75 ± 0.57	5.02 ± 0.68	0.005
Peak gradient, mmHg	91.77 ± 23.01	102.79 ± 28.77	0.006
Mean gradient, mmHg	55.70 ± 16.32	61.43 ± 18.10	0.030
EOA, cm^2^	0.67 ± 0.16	0.65 ± 0.15	0.405
LVEDD, cm	4.87 ± 0.58	4.95 ± 0.71	0.398
LVESD, cm	3.36 ± 0.75	3.32 ± 0.66	0.744
IVS, cm	1.36 ± 0.16	1.37 ± 0.18	0.512
LVPW, cm	1.16 ± 0.14	1.19 ± 0.15	0.182
LV mass, g	244.36 ± 58.71	260.17 ± 81	0.144
LVMI, g/m^2^	142.91 ± 33.52	149.66 ± 42.43	0.247
RV diameter, cm	4.07 ± 0.39	4.04 ± 0.48	0.645
LA diameter, cm	5.05 ± 0.87	4.76 ± 4.76	0.012
RA diameter, cm	4.15 ± 0.60	4.06 ± 0.46	0.259
LVEF, %	55.10 ± 9.51	57.10 ± 6.47	0.108
TAPSE, mm	2.01 ± 0.28	2.03 ± 0.20	0.633

### 3.2 Reverse remodeling at 1 month after AVR

At the 1-month follow-up, peak velocity, peak gradient, and mean gradient were comparable between the SAVR and TAVR groups (Table 3). Compared to baseline, significant improvements in valvular hemodynamics were observed in both groups (Supplementary Tables S1, S2), although the intergroup differences were not statistically significant (all *p* > 0.05, Table 3).

**Table 3:** Post-surgery 1 month —efficacy analysis.

Variables	Post-surgery 1 year			Change from baseline		Difference in LS mean (95% CI)	
	TAVR (*n* = 86)	SAVR (*n* = 87)	*P*-value	TAVR (*n* = 86)	SAVR (*n* = 87)	TAVR - SAVR	*P*-value
Peak velocity, m/s	2.3 (0.39)	2.5 (0.35)	0.727	−2.4 (0.63)	−2.5 (0.74)	0.00 (−0.13, 0.14)	0.946
Peak gradient, mmHg	22.5 (7.54)	25.7 (7.47)	0.809	−69.2 (22.64)	−77.1 (29.18)	0.23 (−2.43, 2.88)	0.867
Mean gradient, mmHg	12.2 (4.29)	14.1 (4.41)	0.867	−43.5 (16.47)	−47.4 (18.50)	0.06 (−1.48, 1.61)	0.938
LVEDD, cm	4.6 (0.40)	4.5 (0.35)	0.411	−0.3 (0.49)	−0.4 (0.58)	0.10 (−0.02, 0.21)	0.108
LVESD cm	3.0 (0.47)	2.9 (0.35)	0.700	−0.4 (0.64)	−0.4 (0.58)	0.05 (−0.09, 0.19)	0.478
IVS, cm	1.2 (0.14)	1.2 (0.12)	0.827	−0.1 (0.15)	−0.2 (0.18)	0.00 (−0.04, 0.04)	0.970
LVPW, cm	1.0 (0.10)	1.0 (0.12)	0.546	−0.1 (0.16)	−0.2 (0.17)	0.01 (−0.03, 0.06)	0.493
LV mass, g	188.3 (37.84)	185.4 (43.73)	0.402	−56.1 (47.73)	−74.8 (62.50)	9.01 (−3.12, 21.13)	0.147
LVMI, g/m^2^	109.3 (22.33)	105.5 (20.15)	0.382	−33.6 (28.59)	−44.1 (35.83)	6.06 (−0.94, 13.05)	0.092
RV diameter, cm	4.0 (0.35)	4.0 (0.30)	0.563	−0.1 (0.42)	−0.0 (0.48)	−0.03 (−0.15, 0.09)	0.606
LA diameter, cm	4.6 (0.78)	4.4 (0.40)	0.120	−0.4 (0.53)	−0.4 (0.46)	0.09 (−0.06, 0.24)	0.253
RA diameter, cm	4.1 (0.51)	4.1 (0.33)	0.384	−0.1 (0.43)	−0.0 (0.40)	0.04 (−0.09, 0.17)	0.524
LVEF, %	59.0 (4.53)	60.2 (2.14)	0.382	3.8 (8.50)	3.1 (6.22)	−0.44 (−1.64, 0.75)	0.470
TAPSE, mm	2.1 (0.21)	2.0 (0.14)	0.165	0.0 (0.30)	−0.0 (0.26)	0.05 (−0.02, 0.12)	0.163

At 1 month postoperatively, the SAVR group demonstrated significantly smaller left ventricular end-diastolic dimension (4.4 ± 0.46 cm vs. 4.7 ± 0.41 cm, *p* = 0.002), left ventricular end-systolic dimension (2.8 ± 0.45 cm vs. 3.0 ± 0.46 cm, *p* = 0.002), right ventricular diameter (3.8 ± 0.34 cm vs. 4.0 ± 0.39 cm, *p* = 0.006), and left atrial diameter (4.3 ± 0.42 cm vs. 4.7 ± 0.75 cm, *p* = 0.006) compared to the TAVR group. No significant differences were observed in interventricular septal thickness, left ventricular posterior wall thickness, LV mass, or LVMI between the two groups (Table 3).

LVEF improved significantly from baseline to 1 month in both the TAVR (55.10% ± 9.51% to 58.81% ± 3.92%, *p* = 0.001) and SAVR groups (57.10% ± 6.47% to 59.34% ± 4.12%, *p* = 0.007; Supplementary Tables S1, S2). However, the 1-month LVEF values were comparable between the groups (59.3% ± 4.15% vs. 58.8% ± 3.93%, *p* = 0.430, Table 3). Tricuspid annular plane systolic excursion (TAPSE) decreased significantly from baseline in the SAVR group (2.03 ± 0.20 cm to 1.93 ± 0.23 cm, *p* = 0.002), whereas no significant change was observed in the TAVR group (2.01 ± 0.28 cm to 2.04 ± 0.18 cm, *p* = 0.412; Supplementary Tables S1, S2).

Efficacy analysis of ventricular remodeling (Table 3) revealed that both groups exhibited reductions in left ventricular end-systolic and end-diastolic dimensions at 1 month, with the SAVR group showing a more pronounced decrease (ΔLVEDD: −0.5 ± 0.65 cm vs. −0.2 ± 0.47 cm, *p* < 0.001; ΔLVESD: −0.6 ± 0.64 cm vs. −0.3 ± 0.55 cm, *p* < 0.001). Left ventricular mass and LVMI decreased from baseline to 1 month in both cohorts, with a significantly greater reduction in the SAVR group (ΔLVM: −67.3 ± 59.31 g vs. −38.2 ± 46.49 g, *p* = 0.003; ΔLVMI: −39.1 ± 33.93 g/m^2^ vs. −22.5 ± 27.08 g/m^2^, *p* = 0.005). Although LVEF improved in both groups at 1 month, the degree of improvement was similar between SAVR and TAVR patients (ΔLVEF: 2.2% ± 6.19% vs. 3.7% ± 8.10%, *p* = 0.288). Finally, no significant change in TAPSE was observed after TAVR, whereas a significant decrease was noted from baseline to 30 days after SAVR (ΔTAPSE: −0.1 ± 0.31 cm vs. 0.0 ± 0.33 cm, *p* < 0.001).

### 3.3 Reverse remodeling at 1 year after AVR

At the 1-year follow-up, significant improvements from baseline were observed in peak velocity, peak gradient, and mean gradient (all *p* < 0.001; Supplementary Tables S1, S2). Both groups demonstrated significant reductions in left ventricular end-diastolic dimension (TAVR: 4.87 ± 0.58 cm to 4.56 ± 0.40 cm, *p* < 0.001; SAVR: 4.95 ± 0.71 cm to 4.52 ± 0.34 cm, *p* < 0.001) and left ventricular end-systolic dimension (TAVR: 3.36 ± 0.75 cm to 2.97 ± 0.47 cm, *p* < 0.001; SAVR: 3.32 ± 0.66 cm to 2.93 ± 0.34 cm, *p* < 0.001) at 1 year postoperatively (Supplementary Tables S1, S2). However, in contrast to the findings at 1 month, no significant between-group differences were observed in the extent of these reductions at 1 year (LVDd: *p* = 0.108; LVDs: *p* = 0.478; Table 4).

**Table 4:** Post-surgery 1 year — efficacy analysis.

Variables	Post-surgery 1 month			Change from baseline		Difference in LS mean (95% CI)	
	TAVR (*n* = 86)	SAVR (*n* = 87)	*P*-value	TAVR (*n* = 86)	SAVR (*n* = 87)	TAVR - SAVR	*P*-value
Peak velocity, m/s	2.4 (0.43)	2.5 (0.41)	0.419	−2.3 (0.57)	−2.5 (0.73)	0.11 (−0.04, 0.27)	0.142
Peak gradient, mmHg	24.7 (8.62)	26.0 (8.37)	0.315	−67.1 (21.11)	−76.8 (28.63)	2.58 (−0.49, 5.64)	0.102
Mean gradient, mmHg	13.2 (5.04)	13.6 (4.73)	0.217	−42.5 (15.10)	−47.9 (18.03)	1.66 (−0.09, 3.42)	0.065
LVEDD, cm	4.7 (0.41)	4.4 (0.46)	0.002	−0.2 (0.47)	−0.5 (0.65)	0.28 (0.15, 0.42)	<0.001
LVESD, cm	3.0 (0.46)	2.8 (0.45)	0.002	−0.3 (0.55)	−0.5 (0.63)	0.29 (0.14, 0.43)	<0.001
IVS, cm	1.3 (0.14)	1.3 (0.17)	0.762	−0.1 (0.15)	−0.1 (0.16)	−0.02 (−0.07, 0.03)	0.520
LVPW, cm	1.1 (0.13)	1.1 (0.17)	0.454	−0.1 (0.16)	−0.1 (0.18)	0.02 (−0.03, 0.08)	0.411
LV mass, g	206.2 (43.59)	192.9 (51.35)	0.052	−38.2 (46.49)	−67.3 (59.31)	20.61 (7.08, 34.14)	0.003
LVMI, g/m^2^	120.4 (23.94)	110.6 (26.09)	0.103	−22.5 (27.08)	−39.1 (33.93)	11.30 (3.49, 19.11)	0.005
RV diameter, cm	4.0 (0.39)	3.8 (0.34)	0.006	−0.1 (0.43)	−0.2 (0.46)	0.20 (0.07, 0.33)	0.002
LA diameter, cm	4.7 (0.75)	4.3 (0.42)	0.006	−0.4 (0.56)	−0.4 (0.54)	0.22 (0.05, 0.38)	0.010
RA diameter, cm	4.2 (0.60)	4.1 (0.47)	0.482	−0.1 (0.43)	−0.0 (0.46)	0.02 (−0.12, 0.17)	0.757
LVEF, %	58.8 (3.93)	59.3 (4.15)	0.430	3.7 (8.10)	2.2 (6.19)	0.69 (−0.58, 1.95)	0.288
TAPSE, mm	2.0 (0.29)	1.9 (0.23)	<0.001	0.0 (0.33)	−0.1 (0.31)	0.12 (0.06, 0.18)	<0.001

A continued decrease in left ventricular mass was noted at 1 year, but the changes were not significantly different between the SAVR and TAVR groups (ΔLVM: −74.8 ± 62.50 g vs. −53.6 ± 48.59 g, *p* = 0.147; ΔLVMI: −44.1 ± 35.83 g/m^2^ vs. −33.6 ± 28.59 g/m^2^, *p* = 0.092).

Left ventricular ejection fraction showed sustained improvement at 1 year in both the TAVR (*p* < 0.001, Supplementary Table S1) and SAVR groups (*p* < 0.001, Supplementary Table S2), with no significant difference in the degree of improvement between them (ΔLVEF: +3.8% ± 8.50% vs. +3.1% ± 6.22%, *p* = 0.470; Table 4). Notably, TAPSE in the SAVR group at 1 year was not significantly different from its baseline value (*p* = 0.925, Supplementary Table S2), suggesting that the right ventricular functional impairment observed at 1 month was transient.

### 3.4 Clinical outcomes

At baseline, a greater proportion of patients in the TAVR cohort (*n* = 18, 20.9%) had severe pulmonary hypertension (mean pulmonary artery pressure ≥45 mmHg) compared to the SAVR cohort (*n* = 11, 12.6%), although this difference was not statistically significant [32 (27, 42) vs. 30 (27, 37), *p* = 0.256] (Table 5). Pulmonary artery pressure improved in both cohorts at the 1-month and 1-year follow-ups. While no significant inter-group difference was observed at 1 month, the TAVR group exhibited significantly higher pulmonary artery pressures at 1 year [30.0 (27.0, 33.0) vs. 27.0 (25.0, 30.0), *p* < 0.001].

**Table 5:** Clinical outcomes and paravalvular aortic regurgitation.

Characteristic	Surgery		*p*-value
	TAVR *N* = 86	SAVR *N* = 87	
Baseline PAP, Median (Q1, Q3)	32 (27, 42)	30 (27, 37)	0.256
1 month PAP, Median (Q1, Q3)	28 (26, 33)	28 (25, 31)	0.121
1 year PAP, Median (Q1, Q3)	30.0 (27.0, 33.0)	27.0 (25.0, 30.0)	<0.001
Discharge paravalvular aortic regurgitation, *n* (%)			
None/Trace	72 (83.7%)	82 (94.3%)	0.035
Mild	13 (15.1%)	5 (5.7%)	
Moderate	1 (1.2%)	0 (0.0%)	
1 month paravalvular aortic regurgitation, *n* (%)			
None/Trace	71 (82.6%)	80 (92.0%)	0.093
Mild	13 (15.1%)	7 (8.0%)	
Moderate	2 (2.3%)	0 (0.0%)	
1 year paravalvular aortic regurgitation, *n* (%)			
None/Trace	51 (59.3%)	68 (78.2%)	<0.001
Mild	12 (14.0%)	15 (17.2%)	
Moderate	23 (26.7%)	4 (4.6%)	
1 month MACE, *n* (%)			
Yes	4 (4.8%)	3 (3.7%)	>0.999
No	79 (95.2%)	79 (96.3%)	
Lost	3	5	
1 year MACE, *n* (%)			
Yes	6 (9.2%)	0 (0.0%)	0.010
No	59 (90.8%)	73 (100.0%)	
Lost	21	14	

PAP, pulmonary artery pressure; MACE, major adverse cardiac events.

Paravalvular regurgitation (PVR) occurred to varying degrees after aortic valve replacement in both cohorts. The severity of PVR was significantly greater in the TAVR cohort both at discharge (*p* = 0.035) and at 1 year (*p* < 0.001), whereas no significant difference was found between the cohorts at 1 month (Table 5). A comparison of PVR severity between the 1-month and 1-year time points revealed an increase in the number of patients with moderate regurgitation, particularly within the TAVR group (increasing from 2.3% to 26.7%).

After excluding patients lost to follow-up, we analyzed the incidence of Major Adverse Cardiac Events (MACE). The results indicated that the TAVR group experienced MACE more frequently at the 1-year follow-up (*p* = 0.01), while no statistically significant difference was observed at 1 month (Table 5). Although a lower incidence of Major Adverse Cardiac Events (MACE) was observed in patients with more pronounced postoperative reverse left ventricular remodeling, this association did not reach statistical significance (*p* = 0.700 at 1 month and *p* = 0.423 at 1 year, Table 6).

**Table 6:** The relationship between ventricular reverse remodeling and clinical outcomes.

Characteristic	Reverse remodeling		*p*-value
	Apparent *N* = 72	Inapparent *N* = 101	
1 month MACE, *n* (%)			
Yes	2 (2.9%)	5 (5.3%)	0.700
No	68 (97.1%)	90 (94.7%)	
1 year MACE, *n* (%)			
Yes	2 (2.8%)	4 (6.2%)	0.423
No	70 (97.2%)	61 (93.8%)	

Fisher's exact test.

**Figure 2:**
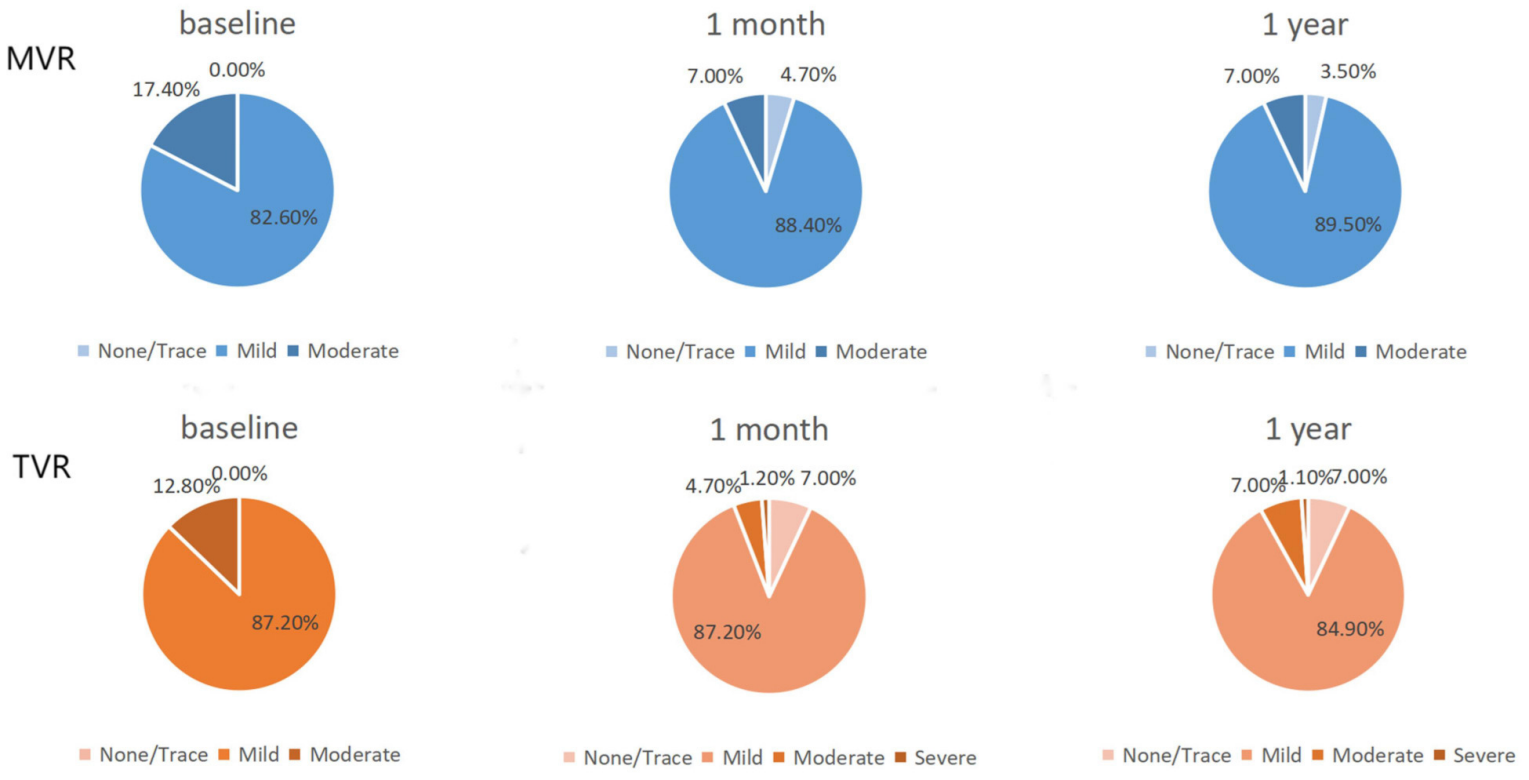
TAVR group other valve improvements. MVR, mitral valve regurgitation; TVR, tricuspid valve regurgitation.

Following the procedure, the severity of mitral and tricuspid valve regurgitation showed significant improvement in the TAVR cohort at both the 1-month and 1-year follow-ups (Figure 2) (all *p*-values <0.001, Supplementary Table S3). In contrast, although there was a trend towards improvement in the degree of other valve regurgitation in the SAVR cohort, the changes did not reach statistical significance (Supplementary Table S3).

## 4 Discussion

This retrospective study compared the characteristics of ventricular remodeling at different time points following SAVR vs. TAVR, providing imaging-based evidence to inform clinical decision-making. To more precisely analyze the impact of aortic valve replacement on ventricular remodeling, we excluded patients who underwent concurrent mitral or tricuspid valve surgery (*n* = 31) but included those who had concomitant aortic root surgery (*n* = 6) or coronary artery bypass grafting (*n* = 21). The results demonstrated that although the TAVR group was older, had more comorbidities, and worse baseline cardiac function, both procedures promoted left ventricular reverse remodeling, with comparable benefits observed at the 1-year follow-up. Notably, given the prior suggestion by F. Sousa Nunes that significant LV reverse remodeling is evident at the earliest follow-up point (1 month post-AVR) (8), we utilized efficacy analysis to compare intergroup differences. We found that SAVR was associated with a more pronounced reduction in left ventricular end-systolic dimension, end-diastolic dimension, and mass in the early postoperative period (30 days). This suggests that SAVR may facilitate a more complete and rapid reduction in afterload compared to TAVR, which is consistent with echocardiographic findings from previous RCTs (9, 10).

However, the observed higher frequency of MACE in the TAVR group at the 1-year follow-up was somewhat unexpected and appears inconsistent with conventional experience and expectations. In addition to the loss of patients to follow-up for whom clinical outcomes could not be determined, this finding should be considered in the context of the TAVR cohort's older age and poorer baseline cardiac function. The relationship between postoperative ventricular remodeling and clinical outcomes remains uncertain at present. The lower pulmonary artery pressure observed in the SAVR cohort at 1 year may be associated with improved left ventricular diastolic function; however, this requires confirmation through longer-term follow-up.

Aortic stenosis causes chronic pressure overload, leading to left ventricular concentric hypertrophy and myocardial fibrosis. AVR alleviates this obstruction, significantly reducing afterload and creating conditions for myocardial reverse remodeling (2, 13, 14). Based on our results, the pattern of post-procedural ventricular remodeling improvement may differ between SAVR and TAVR. This early discrepancy could stem from transient myocardial stretching during balloon valvuloplasty in TAVR (15), or differences in residual pressure gradients and valvular hemodynamics.

The transient right ventricular dysfunction observed at 1 month in the SAVR group (which recovered by 1 year) has also been reported in recent studies (9, 10). This pattern may reflect the more invasive nature of open-heart surgery, involving cardiopulmonary bypass and cardiac manipulation, which can temporarily impact RV function (16). Although recovery at 1 year indicates its reversible nature, the PARTNER 3 trial preliminarily suggested that a decrease in TAPSE at 30 days is associated with adverse outcomes (9). A similar pattern of RV functional changes is seen in other cardiac surgeries requiring cardiopulmonary bypass (17), potentially due to mechanisms like myocardial stunning, systemic inflammatory response, or altered ventricular interdependence following LV remodeling.

SAVR has long been the standard treatment for severe AS until TAVR demonstrated lower mortality in patients at the highest surgical risk, leading to its recommendation in the 2014 AHA/ACC guidelines for this group (18). The 2017 focused update of the AHA/ACC guidelines expanded the evidence of benefit and non-inferiority, extending TAVR use to intermediate-risk patients (19). Recent studies indicate potential benefits of TAVR even in low-risk patients (9). Despite SAVR showing superior early reverse remodeling, TAVR holds promise for favorable long-term outcomes. The primary advantage of SAVR lies in its long-term valve durability. Consequently, based on lifetime management strategy of the valve prosthesis, younger, low-surgical-risk patients often still prefer SAVR.

In summary, this study provides preliminary evidence that SAVR is associated with more pronounced early left ventricular reverse remodeling compared to TAVR. Our findings highlight a potential clinical trade-off: SAVR offers a more potent impetus for left ventricular recovery at the cost of transient right ventricular dysfunction. This knowledge can inform personalized therapeutic strategies. SAVR may be preferable for healthier patients in whom rapid left ventricular recovery is prioritized, while the less invasive nature of TAVR might be advantageous for those less tolerant of peri-procedural right ventricular disturbance. Ultimately, the clinical decision should be comprehensive, integrating patient age, comorbidities, anatomical characteristics, and personal preferences, while also considering operator experience and center-specific volumes. With ongoing advancements in valve technology, imaging, and surgical expertise, future prospects lie in refining patient selection and tailoring individual treatment strategies to further improve outcomes for patients with severe aortic stenosis.

## 5 Limitations

Although this study provides valuable clinical evidence, several limitations should be acknowledged. First, the retrospective study design is inherently susceptible to selection bias. Despite statistical adjustments for covariates, the potential influence of unmeasured confounding factors cannot be excluded. Second, due to the inability to re-analyze historical echocardiographic data, global longitudinal strain (GLS) measurements could not be provided. Third, the follow-up was limited to one year post-procedure because of excessive data loss with longer follow-up intervals; consequently, the impact of the two procedures on very long-term ventricular remodeling could not be assessed. Finally, the relatively limited sample size, which resulted in insufficient statistical power for subgroup analyses, necessitates validation of the current findings in larger, prospective studies.

Furthermore, the echocardiographic parameters measured in this study, while providing valuable insights into ventricular remodeling, represent only a part of the complex structural and functional changes occurring after AVR. More sophisticated measures, such as global longitudinal strain, may offer additional prognostic information and detect subtle improvements in myocardial function that precede changes in conventional parameters (20, 21). Recent technological advances, including wearable acoustic cardiography devices capable of measuring electromechanical activation time, show promise for the continuous monitoring of remodeling progression outside traditional clinical settings (22). The correlation between such novel parameters and established echocardiographic measures of remodeling deserves further investigation in larger prospective studies.

## 6 Conclusion

In patients with severe aortic stenosis, SAVR was associated with more significant regression of left ventricular dimensions and mass at 1 month compared to TAVR, alongside a transient impairment of right ventricular function. By 1 year postoperatively, however, no significant differences in ventricular remodeling were observed between the two groups.

## Data Availability

The data analyzed in this study is subject to the following licenses/restrictions: The dataset used in this study was sourced from the Hospital Information System (HIS) of The Affiliated Hospital of Qingdao University. In accordance with the ethical approval (Approval No.: QYFY WZLL 29907) granted by the hospital's Ethics Committee and to protect patient privacy and confidentiality, access to the raw data is restricted. However, upon reasonable request, the de-identified dataset (including baseline characteristics, echocardiography-related data, etc.) collected and organized by the researchers for this study can be made available to interested researchers. Requests to access these datasets should be directed to Haoyan Li, honoka138964@163.com.
