# Supplementary material for: Comparative study of transcatheter aortic valve implantation versus conventional surgical aortic valve replacement in the treatment of severe aortic stenosis with reverse ventricular remodeling
Source: Front Cardiovasc Med. 2026 Jan 6;12:1712400. doi: 10.3389/fcvm.2025.1712400 (PMC12816305; doi:10.3389/fcvm.2025.1712400)
Supplement: Supplementary file 1 [file Datasheet1.pdf]

Additional materials

Table 1 Changes in Echocardiographic Parameters from Baseline to Post-TAVR

TAVR	Baseline	Post-surgery 1month	P-value	Post-surgery 1year	P-value
Peak velocity, m/s	4.75 (0.57)	2.44 (0.43)	<0.001	2.33 (0.38)	<0.001
Peak gradient, mmHg	91.78 (23.02)	24.72 (8.61)	<0.001	22.54 (7.53)	<0.001
Mean gradient, mmHg	55.71 (16.32)	13.02 (5.03)	<0.001	12.17 (4.28)	<0.001
LVEDD, cm	4.87 (0.58)	4.65 (0.41)	0.006	4.56 (0.40)	<0.001
LVESD, cm	3.36 (0.75)	3.03 (0.45)	0.001	2.97 (0.47)	<0.001
IVS, cm	1.36 (0.16)	1.27 (0.14)	<0.001	1.23 (0.12)	<0.001
LVPW, cm	1.16 (0.14)	1.08 (0.13)	<0.001	1.03 (0.09)	<0.001
LV mass, g	244.36 (58.71)	206.20 (43.59)	<0.001	188.31 (37.83)	<0.001
LVMI, g/m2	142.91 (33.52)	120.40 (23.93)	<0.001	109.29 (22.32)	<0.001
RV diameter, cm	4.07 (0.39)	3.96 (0.38)	0.070	3.96 (0.35)	0.073
LA diameter, cm	5.05 (0.87)	4,67 (0.75)	0.003	4,60 (0.77)	<0.001
RA diameter, cm	4.15 (0.60)	4.06 (0.55)	0.301	4.11 (0.50)	0.616
LVEF, %	55.10 (9.51)	58.81 (3.92)	0.001	58.95 (4.52)	<0.001
TAPSE, mm	2.01 (0.28)	2.04 (0.18)	0.412	2.05 (0.21)	0.378

Mean(SD)

Table 2 Changes in Echocardiographic Parameters from Baseline to Post-SAVR

SAVR	Baseline	Post-surgery 1month	P-value	Post-surgery 1year	P-value
Peak velocity, m/s	5.02 (0.68)	2.52 (0.41)	<0.001	2.50 (0.35)	<0.001
Peak gradient, mmHg	102.79 (28.77)	26.02 (8.36)	<0.001	25.73 (7.46)	<0.001
Mean gradient, mmHg	61.44 (18.10)	13.56 (4.73)	<0.001	14.07 (4.40)	<0.001
LVEDD, cm	4.95 (0.71)	4.40 (0.46)	<0.001	4.52 (0.34)	<0.001
LVESD, cm	3.32 (0.66)	2.77 (0.45)	<0.001	2.93 (0.34)	<0.001
IVS, cm	1.37 (0.18)	1.29 (0.17)	0.001	1.22 (0.13)	<0.001
LVPW, cm	1.19 (0.15)	1.08 (0.17)	<0.001	1.01 (0.11)	<0.001
LV mass, g	260.17 (81.01)	192.85 (51.35)	<0.001	185.37 (43.73)	<0.001
LVMI, g/m2	149.66 (42.43)	110.60 (26.08)	<0.001	105.54 (20.14)	<0.001
RV diameter, cm	4.04 (0.48)	3.82 (0.34)	<0.001	4.00 (0.29)	0.547
LA diameter, cm	4.76 (0.58)	4.34 (0.42)	<0.001	4.40 (0.40)	<0.001
RA diameter, cm	4.06 (0.46)	4.02 (0.41)	0.581	4.08 (0.33)	0.685
LVEF, %	57.10 (6.47)	59.34 (4.12)	0.007	60.24 (2.13)	<0.001
TAPSE, mm	2.03 (0.20)	1.93 (0.23)	0.002	2.03 (0.14)	0.925

Mean(SD)

Table3 Other valve improvements After Treatment

	TAVR				SAVR			
	1month	1year	Δ BL-1month *P*	Δ BL-1year *P*	1month	1year	Δ BL-1month *P*	Δ BL-1year *P*
MV regurgitation, %	N=86	N=86	<0.001	<0.001	N=87	N=87	0.215	0.644
None/Trace	4.7%	3.5%			14.9%	8.0%		
Mild	88.4%	89.5%			85.1%	90.8%		
Moderate	7.0%	7.0%			0.0%	1.1%		
Severe	0.0%	0.0%			0.0%	0.0%		
TV regurgitation,%	N=86	N=86	<0.001	<0.001	N=87	N=87	0.105	0.715
None/Trace	7.0%	7.0%			17.2%	10.3%		
Mild	87.2%	84.9%			80.5%	87.4%		
Moderate	4.7%	7.0%			2.3%	2.3%		
Severe	1.2%	1.1%			0.0%	0.0%		
